# 2078- Immunotherapy – 2078. Allergen-specific oral immunotherapy for peanut allergy: a Cochrane systematic review

**DOI:** 10.1186/1939-4551-6-S1-P160

**Published:** 2013-04-23

**Authors:** Ulugbek Nurmatov, Iris Venderbosch, Graham Devereux, F Estelle R Simons, Aziz Sheikh

**Affiliations:** 1 Allergy and Respiratory Research Group, Centre for Population Health Sciences, The University of Edinburgh, Edinburgh, UK; 2 Radboud University Nijmegen Medical Center, Nijmegen, Netherlands; 3 Department of Child Health, Royal Aberdeen Children's Hospital, The University of Aberdeen, Aberdeen, UK; 4 Pediatrics and Child Health, Immunology, University of Manitoba, Winnipeg, Canada

## Background

Allergen-specific oral immunotherapy (OIT) aims to induce desensitisation and immune tolerance, which should if successful reduce the risk of further reactions to peanuts and peanut-containing foods.

## Methods

We performed a systematic review of intervention studies by searching 13 databases and contacting an international panel of experts. Studies were critically appraised using Cochrane criteria.

## Results

We identified one RCT with 28 children age 1-16 years (19 in the OIT group and nine in the placebo group). Because of allergic side-effects, three children were withdrawn from the OIT group early in the study. The remaining 16 participants in the OIT group completed the study and ingested a maximum cumulative dose (MCD) of 5000 mg (≈20 peanuts). All 9 participants in the placebo group completed the study, but ingested an MCD of only 280 mg (range, 0-1900 mg, P<0.001). Children in the OIT group had reductions in peanut-specific skin prick tests (P<0.001), IL-5 (P=0.01), and IL-13 (P=0.02), and increases in peanut-specific IgG4 (P<0.01) and T reg cells. Nine children (47%) of the 19 in the OIT group experienced side-effects and two of them required epinephrine treatment.

## Conclusions

We found one small RCT judged to be at low risk of bias which showed that peanut OIT can result in desensitisation in children, and that this is associated with evidence of concurrent immune-modulation. This treatment approach was however associated with substantial risk of adverse reactions, although most of these were mild. Thus, peanut OIT cannot currently be recommended as a treatment for the management of patients with IgE-mediated peanut allergy. Larger RCTs are needed investigating the acceptability, effectiveness and cost-effectiveness of safer treatment regimens, particularly in relation to the induction of long-term immune tolerance.

